# Supplementary material for: Evaluating the reproducibility and verifiability of nutrition research: a case study of studies assessing the relationship between potatoes and colorectal cancer
Source: Nutr Diabetes. 2026 Jun 23;16:35. doi: 10.1038/s41387-026-00444-8 (PMC13542112; doi:10.1038/s41387-026-00444-8)
Supplement: Supplementary file 1 [file 41387_2026_444_MOESM1_ESM.pdf]

Evaluating the Reproducibility and Verifiability of Nutrition Research: A Case Study of Studies Assessing the Relationship Between Potatoes and Colorectal Cancer

Yasaman Jamshidi-Naeini [1], Colby J. Vorland [1], Pranav Kapoor [1], Bailey Ortyl [1], Jocelyn Mineo [1], Luke Still [1], Korlu Sorsor [1], Shelby Rodney [1], Xander Tooze [1], Brent Flickinger [2], Beate Henschel [1], Stephanie L. Dickinson [1], David B. Allison [1]*

[1] Department of Epidemiology and Biostatistics, Indiana University School of Public Health-Bloomington, IN, USA

[2] Department of Applied Health Science, Indiana University School of Public Health-Bloomington, IN, USA

* Correspondence: David B. Allison, allison@iu.edu. 1025 E. 7th St. PH 111. 812-855-1250.

Appendix 1

PubMed Search String

potato*[tiab] OR solanum tuberosum[tiab] OR french fry[tiab] OR french fries[tiab] OR potato chip*[tiab]
colon[tiab] OR rectum[tiab] OR rectal[tiab] OR colorectal[tiab] OR colorectum[tiab] OR bowel[tiab]
prospective OR cohort OR longitudinal OR follow-up OR case-cohort OR nested case-control
(#1 AND #2 AND #3)

Scopus Search String

TITLE-ABS ((potato*) OR ("solanum tuberosum") OR ("french fry") OR ("french fries") OR ("potato chip*"))
AND
TITLE-ABS ((colon) OR (rectum) OR (rectal) OR (colorectal) OR (colorectum) OR (bowel))
AND
((prospective) OR (cohort) OR (longitudinal) OR (follow-up) OR (case-cohort) OR ("nested case-control"))

Web of Science Search String

TS=((potato*) OR ("solanum tuberosum") OR ("french fry") OR ("french fries") OR ("potato chip*"))
AND
TS=((colon) OR (rectum) OR (rectal) OR (colorectal) OR (colorectum) OR (bowel))
AND
TS=((prospective) OR (cohort) OR (longitudinal) OR (follow-up) OR (case-cohort) OR ("nested case-control"))

Appendix 2

Reproducibility analysis of Flood et al. (2002)

This is documentation of our attempts in reproducing tables 2 and 3 from Flood et al., doi:[10.1093/ajcn/75.5.936](https://doi.org/10.1093/ajcn/75.5.936)

1. Sample Size

1. Flood et al 2002 indicated in the table footnotes that 45,490 participants were used in the analysis
    - If we manually use the exclusions listed in the paper, we obtain **45,494** where discrepancies seem to be due to redacted dates that prohibited identification of 4 ineligible participants.
    - We could obtain the number **45,490** for sample size after using variable 'exc_fl' that was specifically made to categorize the exclusions and excluding those with >16 servings of fruit and/or veggies per day.
2. One place in the text mentioned **41,323** had 'complete' study data which is a discrepancy from the 45,490 indicated in the table footnotes.
    - Using same criteria, we manually obtained **41,219**.

2. Fruit and Vegetable Intake Quintiles

3. Table 2 calls for quintiles of fruit and vegetable intake in servings per day per 1000 kJ
    - No variables appeared to indicate servings per day in the dataset we received, but it did have frequencies of food items per week:
        - Without a variable for servings, we used frequency of consumption by dividing the weekly frequencies by seven to estimate daily servings.
        - To convert kcal to kJ servings per day, 1 kcal = 4.184 kJ.
        - Divide servings per day by kJ per day, then multiply by 1000 kJ

TABLE 2
Baseline characteristics of the Breast Cancer Detection Demonstration Project follow-up cohort, by quintile of fruit and vegetable intakes[1]

	Quintile of fruit intake					Quintile of vegetable intake				
	1 (lowest)	2	3	4	5 (highest)	1 (lowest)	2	3	4	5 (highest)
Fruit intake (servings/d)										
Median	0.05	0.14	0.22	0.32	0.50	—	—	—	—	—
Cutoff	<0.10	<0.18	<0.26	<0.38	≥0.38	—	—	—	—	—
Vegetable intake (servings/d)										
Median	—	—	—	—	—	0.25	0.40	0.52	0.68	0.98
Cutoff	—	—	—	—	—	<0.33	<0.46	<0.59	<0.79	≥0.79

Our Results for Median Values of Fruit and Vegetable Intake by Quintile

Quintile	1	2	3	4	5
Fruit	0.05	0.14	0.23	0.33	0.52
Vegetable	0.21	0.33	0.43	0.56	0.81

Our Results for Quintile Cutoff:

Variable	20th Pctl	40th Pctl	60th Pctl	80th Pctl
fruit_se_per_kcal	0.10	0.19	0.28	0.40
veg_se_per_kcal	0.29	0.40	0.51	0.67

3. Risk models of Colorectal Cancer by F&V Quintiles

We attempted to reproduce the model Risk estimates for nutrient-density models (Table 3 "Energy-adjusted model").

A. ***Try 1:*** Using the cutoffs defined in Table 2 of the paper:

```
*try models with quintiles in Table 2;
data floodreal;
    set floodreal;
    if fruit_se_per_kcal < 0.10 then fruit_quint = 1;
    if 0.10 <= fruit_se_per_kcal < 0.18 then fruit_quint = 2;
    if 0.18 <= fruit_se_per_kcal < 0.26 then fruit_quint = 3;
    if 0.26 <= fruit_se_per_kcal < 0.38 then fruit_quint = 4;
    if fruit_se_per_kcal >= 0.38 then fruit_quint = 5;

    if veg_se_per_kcal < 0.33 then veg_quint = 1;
    if 0.33 <= veg_se_per_kcal < 0.46 then veg_quint = 2;
    if 0.46 <= veg_se_per_kcal < 0.59 then veg_quint = 3;
    if 0.59 <= veg_se_per_kcal < 0.79 then veg_quint = 4;
    if veg_se_per_kcal >= 0.79 then veg_quint = 5;

run;
*try primary model;
data floodreal;
    set floodreal;
    fruit_model = fruit_se_per_day / diet_kcal;
    veg_model = veg_se_per_day / diet_kcal;
run;
proc phreg data = floodreal ;
class fruit_quint(ref="1") veg_quint(ref="1") ;
model AGE_CRC_E*crc_dummy(0) = fruit_quint veg_quint fruit_model veg_model diet_kcal / risklimits;
run;
```

Analysis of Maximum Likelihood Estimates

Parameter		DF	Parameter Estimate	Standard Error	Chi-Square	Pr > ChiSq	Hazard Ratio	95% Hazard Ratio Confidence Limits	
fruit_quint	2	1	-0.10540	0.16271	0.4196	0.5171	0.900	0.654	1.238
fruit_quint	3	1	-0.20752	0.17196	1.4564	0.2275	0.813	0.580	1.138
fruit_quint	4	1	0.00961	0.17668	0.0030	0.9566	1.010	0.714	1.427
fruit_quint	5	1	0.09274	0.24267	0.1461	0.7023	1.097	0.682	1.765
veg_quint	2	1	-0.26596	0.13930	3.6453	0.0562	0.766	0.583	1.007
veg_quint	3	1	-0.27842	0.15945	3.0491	0.0808	0.757	0.554	1.035
veg_quint	4	1	-0.17566	0.18318	0.9196	0.3376	0.839	0.586	1.201
veg_quint	5	1	-0.56822	0.28692	3.9219	0.0477	0.567	0.323	0.994
fruit_model		1	-58.09525	88.15091	0.4343	0.5099	0.000	0.000	6.364E49
veg_model		1	103.22099	63.94075	2.6060	0.1065	6.735E44	0.000	1.798E99
diet_kcal		1	-8.9275E-7	0.0000917	0.0001	0.9922	1.000	1.000	1.000

TABLE 3

Energy-adjusted and fully adjusted relative risks (and 95% CIs) for colorectal cancer in the Breast Cancer Detection Demonstration Project follow-up cohort, by quintile (Q) of fruit and vegetable intakes[1]

	Energy-adjusted model[2]	Fully adjusted model[3]
Fruit intake (servings/d)		
Q1, <0.09	1.00 (reference)	1.00
Q2, 0.10–0.17	0.94 (0.70, 1.26)	0.97 (0.72, 1.29)
Q3, 0.18–0.25	0.85 (0.63, 1.15)	0.91 (0.67, 1.23)
Q4, 0.26–0.37	1.07 (0.81, 1.42)	1.14 (0.86, 1.52)
Q5, 0.38	1.09 (0.82, 1.44)	1.15 (0.86, 1.53)
Vegetable intake (servings/d)		
Q1, <0.32	1.00 (reference)	1.00
Q2, 0.33–0.45	0.77 (0.58, 1.02)	0.78 (0.59, 1.04)
Q3, 0.46–0.58	0.83 (0.63, 1.10)	0.86 (0.65, 1.14)
Q4, 0.59–0.78	0.90 (0.69, 1.19)	0.93 (0.70, 1.22)
Q5, 0.79	0.92 (0.70, 1.22)	0.95 (0.71, 1.26)

[1] $n = 45490$. All intakes are per 1000 kJ.

[2] Used the nutrient-density method with energy in the model to adjust for total energy intake.

Try 1 with removing fruit_model and veg_model [1] from the model

Analysis of Maximum Likelihood Estimates									
Parameter		DF	Parameter Estimate	Standard Error	Chi-Square	Pr > ChiSq	Hazard Ratio	95% Hazard Ratio Confidence Limits	
fruit_quint	2	1	-0.12231	0.15914	0.5906	0.4422	0.885	0.648	1.209
fruit_quint	3	1	-0.24572	0.15987	2.3622	0.1243	0.782	0.572	1.070
fruit_quint	4	1	-0.05339	0.14652	0.1328	0.7156	0.948	0.711	1.263
fruit_quint	5	1	-0.03527	0.14451	0.0596	0.8072	0.965	0.727	1.281
veg_quint	2	1	-0.19613	0.13289	2.1782	0.1400	0.822	0.633	1.066
veg_quint	3	1	-0.15330	0.14049	1.1907	0.2752	0.858	0.651	1.130
veg_quint	4	1	0.01596	0.14122	0.0128	0.9100	1.016	0.770	1.340
veg_quint	5	1	-0.20573	0.16498	1.5551	0.2124	0.814	0.589	1.125
diet_kcal		1	-7.5676E-6	0.0000915	0.0068	0.9341	1.000	1.000	1.000

Try 2: Using quintile cutoffs we obtained [calculated in SAS (fruit_se_per_kcal & veg_se_per_kcal)]

Analysis of Maximum Likelihood Estimates									
Parameter		DF	Parameter Estimate	Standard Error	Chi-Square	Pr > ChiSq	Hazard Ratio	95% Hazard Ratio Confidence Limits	
fruit_quint	2	1	-0.13215	0.16028	0.6798	0.4097	0.876	0.640	1.200
fruit_quint	3	1	-0.13841	0.16961	0.6659	0.4145	0.871	0.624	1.214
fruit_quint	4	1	0.05338	0.18580	0.0825	0.7739	1.055	0.733	1.518
fruit_quint	5	1	0.07778	0.26046	0.0892	0.7652	1.081	0.649	1.801
veg_quint	2	1	-0.15546	0.15767	0.9721	0.3242	0.856	0.628	1.166
veg_quint	3	1	-0.27499	0.16688	2.7153	0.0994	0.760	0.548	1.053
veg_quint	4	1	-0.18942	0.17944	1.1143	0.2912	0.827	0.582	1.176
veg_quint	5	1	-0.17175	0.24436	0.4940	0.4821	0.842	0.522	1.360
fruit_model		1	-57.29595	92.22917	0.3859	0.5344	0.000	0.000	4.191E53
veg_model		1	26.40572	65.36014	0.1632	0.6862	2.937E11	0.000	1.266E67
diet_kcal		1	1.19565E-6	0.0000915	0.0002	0.9896	1.000	1.000	1.000

[1] It is mentioned in the original paper by Flood et al. that "the primary model was the multivariate nutrient-density model, in which servings of fruit or vegetables divided by total energy entered the model along with total energy (as a separate covariate)." fruit_model is the servings of fruit divided by the total energy in kilocalories and the veg_model is the servings of veggies divided by kilocalories. As including the servings of fruits and vegetables (divided by calories) in addition to the quintiles which were also defined by servings of fruits and vegetables in the model was an unfamiliar approach to us, we did an exploratory analysis without fruit_model and veg_model variables.

Try 2 with removing fruit_model and veg_model from model:

Analysis of Maximum Likelihood Estimates									
Parameter		DF	Parameter Estimate	Standard Error	Chi-Square	Pr > ChiSq	Hazard Ratio	95% Hazard Ratio Confidence Limits	
fruit_quint	2	1	-0.15500	0.15583	0.9894	0.3199	0.856	0.631	1.162
fruit_quint	3	1	-0.18356	0.15344	1.4311	0.2316	0.832	0.616	1.124
fruit_quint	4	1	-0.01635	0.14879	0.0121	0.9125	0.984	0.735	1.317
fruit_quint	5	1	-0.05586	0.14853	0.1414	0.7069	0.946	0.707	1.265
veg_quint	2	1	-0.13936	0.15360	0.8232	0.3643	0.870	0.644	1.175
veg_quint	3	1	-0.24687	0.15447	2.5540	0.1100	0.781	0.577	1.057
veg_quint	4	1	-0.14607	0.15010	0.9470	0.3305	0.864	0.644	1.160
veg_quint	5	1	-0.09248	0.14947	0.3828	0.5361	0.912	0.680	1.222
diet_kcal		1	-1.2594E-6	0.0000914	0.0002	0.9890	1.000	1.000	1.000

B. ***Try 3:*** Using cutoffs defined in Table 3 of paper:

- Note that there is an internal inconsistency in the paper which appears to define the cutoffs slightly differently in Table 2 and Table 3.

Analysis of Maximum Likelihood Estimates									
Parameter		DF	Parameter Estimate	Standard Error	Chi-Square	Pr > ChiSq	Hazard Ratio	95% Hazard Ratio Confidence Limits	
fruit_quint	2	1	-0.10570	0.16271	0.4220	0.5159	0.900	0.654	1.238
fruit_quint	3	1	-0.20749	0.17194	1.4563	0.2275	0.813	0.580	1.138
fruit_quint	4	1	0.01017	0.17663	0.0033	0.9541	1.010	0.715	1.428
fruit_quint	5	1	0.09480	0.24256	0.1528	0.6959	1.099	0.683	1.769
veg_quint	2	1	-0.26949	0.13907	3.7550	0.0527	0.764	0.582	1.003
veg_quint	3	1	-0.26719	0.16014	2.7840	0.0952	0.766	0.559	1.048
veg_quint	4	1	-0.20660	0.17927	1.3280	0.2492	0.813	0.572	1.156
veg_quint	5	1	-0.58740	0.28433	4.2680	0.0388	0.556	0.318	0.970
fruit_model		1	-58.28093	88.08551	0.4378	0.5082	0.000	0.000	4.65E49
veg_model		1	108.07238	62.77915	2.9635	0.0852	8.615E46	0.000	2.36E100
diet_kcal		1	-2.014E-6	0.0000918	0.0005	0.9825	1.000	1.000	1.000

Try 3 with removing fruit_model and veg_model from the model

Analysis of Maximum Likelihood Estimates									
Parameter		DF	Parameter Estimate	Standard Error	Chi-Square	Pr > ChiSq	Hazard Ratio	95% Hazard Ratio Confidence Limits	
fruit_quint	2	1	-0.12274	0.15915	0.5948	0.4406	0.884	0.647	1.208
fruit_quint	3	1	-0.24580	0.15987	2.3638	0.1242	0.782	0.572	1.070
fruit_quint	4	1	-0.05303	0.14652	0.1310	0.7174	0.948	0.712	1.264
fruit_quint	5	1	-0.03368	0.14450	0.0543	0.8157	0.967	0.728	1.283
veg_quint	2	1	-0.19650	0.13289	2.1865	0.1392	0.822	0.633	1.066
veg_quint	3	1	-0.13858	0.14265	0.9438	0.3313	0.871	0.658	1.151
veg_quint	4	1	-0.00900	0.13927	0.0042	0.9485	0.991	0.754	1.302
veg_quint	5	1	-0.20709	0.16497	1.5757	0.2094	0.813	0.588	1.123
diet_kcal		1	-9.0276E-6	0.0000915	0.0097	0.9214	1.000	1.000	1.000

- In all three attempts, we obtained results in the same ball-park as those in the table (for fruit), but not for vegetables and for RRs in Table 3.
- Note that we obtain Hazard Ratios which are standard from the Survival analysis. We are not confident if these have been converted to Risk Ratios (RRs) in the paper, or they mean HRs.
- We also are not confident whether Flood et al. ran separate models for fruits and vegetables or included in the same model. We tried either way, and did not obtain the same numerical values as those published.
